# PIWI‐interacting RNAs and human testicular function

**DOI:** 10.1002/wsbm.1572

**Published:** 2022-07-19

**Authors:** Gülizar Saritas, Ailsa Maria Main, Sofia Boeg Winge, Nina Mørup, Kristian Almstrup

**Affiliations:** ^1^ The Department of Growth and Reproduction Copenhagen University Hospital Copenhagen Denmark; ^2^ International Center for Research and Research Training in Endocrine Disruption of Male Reproduction and Child Health (EDMaRC) Copenhagen Denmark; ^3^ The Department of Cellular and Molecular Medicine Faculty of Health and Medical Sciences, University of Copenhagen Copenhagen Denmark

**Keywords:** biomarkers, piRNAs, spermatogenesis

## Abstract

Small noncoding RNAs (sncRNAs) are pieces of RNA with a length below 200 bp and represent a diverse group of RNAs having many different biological functions. The best described subtype is the microRNAs which primarily function in posttranscriptional gene regulation and appear essential for most physiological processes. Of particular interest for the germline is the PIWI‐interacting RNAs (piRNAs) which are a class of sncRNA of 21–35 bp in length that are almost exclusively found in germ cells. Recently, it has become clear that piRNAs are essential for testicular function, and in this perspective, we outline the current knowledge of piRNAs in humans. Although piRNAs appear unique to germ cells, they have also been described in various somatic cancers and biofluids. Here, we discuss the potential function of piRNAs in somatic tissues and whether detection in biofluids may be used as a biomarker for testicular function.

This article is categorized under:

Reproductive System Diseases > Genetics/Genomics/Epigenetics
Reproductive System Diseases > Molecular and Cellular Physiology

## 1 INTRODUCTION

Noncoding RNAs (ncRNAs) are RNAs that are not translated into proteins. There are many different types of ncRNAs, which are commonly divided into long (lncRNAs) and small (sncRNAs) based on a cut‐off of 200 bp (Quinn & Chang, 2016). lncRNAs have been implicated in many different biological and physiological processes (reviewed in Andergassen & Rinn, 2021; Quinn & Chang, 2016) and the number of lncRNAs exceeds the number of protein‐coding genes in the human genome. lncRNAs mainly function as scaffolds regulating, for example, transcriptional access to chromatin or as precursors for sncRNAs (Quinn & Chang, 2016). sncRNAs are also a diverse group of RNAs and are further subdivided into groups based on their mode of action and size as outlined in Table 1.

**TABLE 1:** Subtypes of small noncoding RNAs

Type	Subtype	Approximate size (nt)	Features
Small noncoding RNAs (sncRNAs)	MicroRNA (miRNA)	18–25	Posttranscriptional regulation in most cells
	PIWI‐interacting (piRNA)	21–35	Specific to germ cells
	tRNA‐fragments (tRFs)	15–40	Found in high quantities in the epididymis
	Small nucleolar RNAs (snoRNA)	60–300	RNA processing
	Small nuclear (snRNA)	100–200	Modulate RNA pol II activity
Long noncoding RNAs (lncRNAs)	lncRNA	>200	Scaffolds or precursor for sncRNAs

The most well‐described subtype is the microRNAs (miRNAs), which are involved in posttranscriptional gene regulation. Mostly they suppress gene expression (Ha & Kim, 2014) but miRNAs have also been found to activate gene expression (Vasudevan, 2012), and studies have suggested that miRNAs work across several cellular compartments to control both transcription and the rate of translation (Makarova et al., 2016). While most miRNAs exert their function intracellularly, some are also secreted to the extracellular environment. Hence, miRNAs have been detected in several biofluids including plasma, serum, saliva, tears, urine, breast milk, colostrum, peritoneal fluid, cerebrospinal fluid, bronchial lavage, and seminal and follicular fluid (Sohel, 2016).

In biofluids, miRNAs and other sncRNAs are primarily found in exosomes or associated with binding proteins (O'Brien et al., 2018). There is increasing evidence that the secretion of miRNAs is a regulated process (Bayraktar et al., 2017; Kosaka et al., 2010; O'Brien et al., 2018). Several studies have demonstrated that sncRNAs can act as signaling molecules and play a regulatory role in the recipient cells (Bayraktar et al., 2017; Fabbri, 2018). One example is spermatozoa in mice, which take up exosomes in the epididymis and hence show different sncRNA profiles when testicular and ejaculated spermatozoa are compared (Sharma et al., 2018). Consequently, the most prevalent type of sncRNA in testicular spermatozoa is PIWI‐interacting RNAs (piRNAs), whereas in ejaculated spermatozoa, it is tRNA‐fragments (tRFs) (Sharma et al., 2018). However, it remains unresolved to what degree the sncRNAs found in biofluids represent an endocrine signaling pathway that mirrors the physiological states of the body. Here, we more specifically try to assess the current evidence of whether piRNAs can act as biomarkers for testicular function.

## 2 PIWI‐INTERACTING RNAS

As their name refers to, PIWI‐interacting RNAs (piRNAs) interact with the P‐element‐induced wimpy testis (PIWI; Lin & Spradling, 1997) proteins—a subclade of the Argonaute protein family. Mature piRNAs are 21–35 nt long with a classical 3′ end modification of 2′‐*O*‐methylation (Ozata et al., 2019). In terms of unique sncRNA sequences, piRNAs represent, by far, the largest group of sncRNAs with >181 million unique piRNA sequences from 44 species listed in the database piRBase (Wang et al., 2022). For humans specifically, approximately 8.5 million unique entries exist in piRBase. piRNAs are found in both meiotic and post‐meiotic germ cells of the adult and juvenile testis, and measurement of piRNAs in biofluids could therefore represent a valuable read‐out of testicular function in adult men (Özata et al., 2020).

### 2.1 Function

**FIGURE 1:**
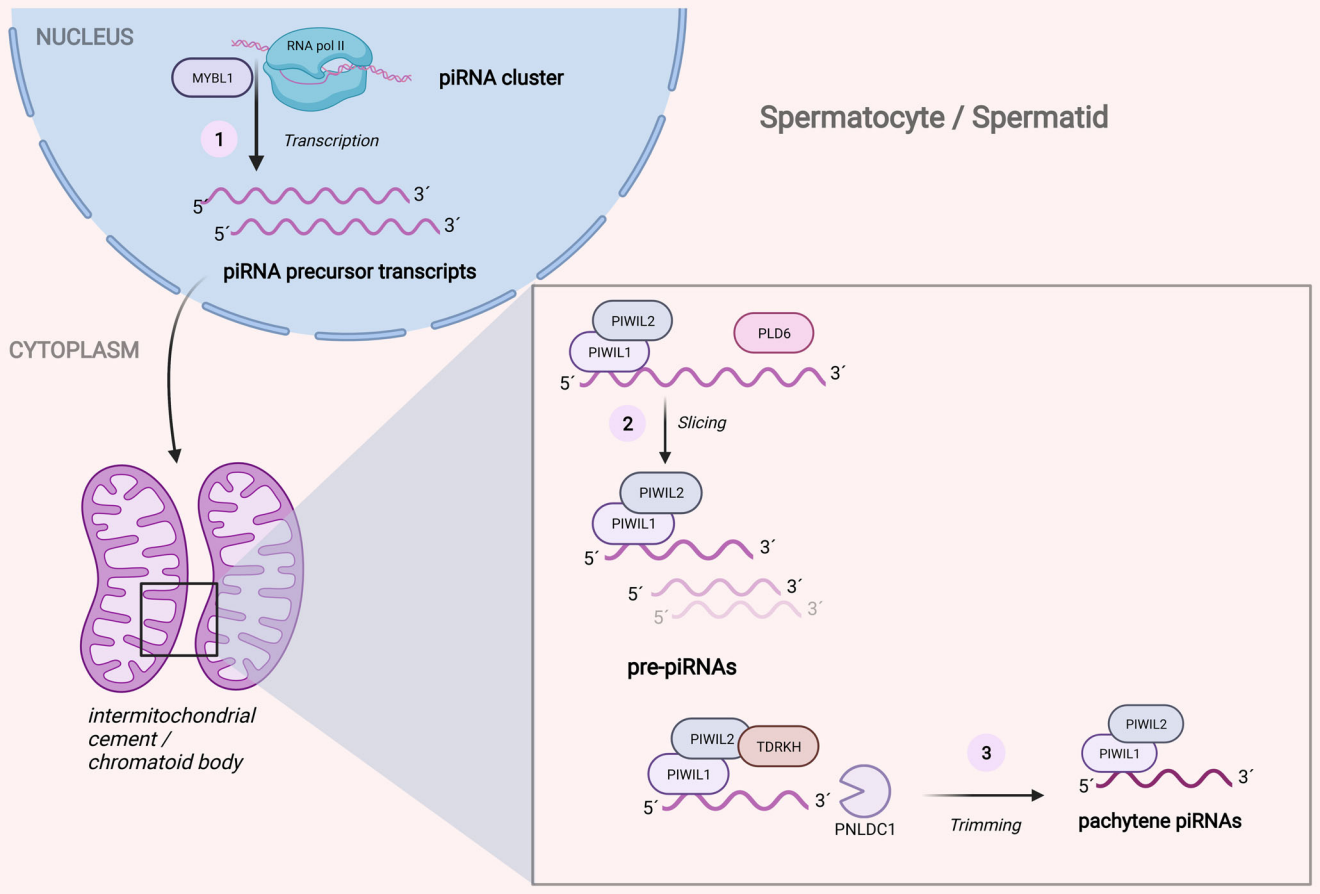
Schematic illustration of pachytene PIWI‐interacting RNA (piRNA) biogenesis in spermatocytes and spermatids. 1) In the nucleus, piRNA precursor transcripts are transcribed from piRNA clusters, which involves RNA pol II and the transcription factor, MYBL1. 2) After cytoplasmic translocation to the intermitochondrial cement or chromatoid body (not illustrated), these precursor transcripts undergo slicing in a process involving PIWIL1, PIWIL2, and PLD6 to produce pre‐piRNAs). 3) Further processing of pre‐piRNAs occurs by PNLDC1 in complex with multiple factors like TDRKH, PIWIL1, and PIWIL2 where PNLDC1 trims the 3′ end. Finally, the 3′ end is modified by HENM1 with a 2′‐*O*‐methylation (not illustrated) forming mature piRNAs associated with PIWIL1 and PIWIL2. Mature piRNAs are important for the completion of spermatogenesis due to their involvement in transcriptional and translational control (see main text). HENM1: HEN methyltransferase 1, MYBL1: MYB proto‐oncogene like 1, piRNA: PIWI‐interacting RNAs, PIWIL1 or 2: PIWI‐like 1 or 2, PLD6: Phospholipase D6, PNLDC1: 3′ exonuclease PARN‐like ribonuclease domain containing 1, RNA pol II: RNA polymerase II, TDRKH: Tudor and KH domain‐containing protein

piRNAs have been described to have different functions depending on what cell type and developmental stage they are expressed in. Hence, piRNAs are commonly further subdivided into fetal/prepachytene and pachytene piRNAs and even further subgroups have been suggested (Sun et al., 2021). In mice, prepachytene piRNAs associate with MILI (PIWIL2 in humans) and MIWI2 (PIWIL4 in humans) during the fetal development of the testis and with MILI in the early stages of spermatogenesis, while pachytene piRNAs associate with MIWI (PIWIL1 in humans) and MILI in pachytene spermatocytes and round spermatids (Iwasaki et al., 2015; Sun et al., 2021) (Figure 1).

#### 2.1.1 Fetal/prepachytene piRNAs

In general, piRNAs found in fetal germ cells are antisense to transposable elements (TEs), and control aberrant expression of TEs during developmental periods of global DNA methylation erasure (Ozata et al., 2019; Sun et al., 2021). If piRNAs did not keep expression of TEs in check, the integrity of the genome would be at risk. PIWIL2 and/or PIWIL4 appear together with prepachytene piRNAs from the stage of gonocytes to pachytene spermatocytes and are both necessary for TE silencing and ping‐pong amplification (secondary piRNA biogenesis) of piRNAs (Iwasaki et al., 2015; Kuramochi‐Miyagawa et al., 2008). Besides mediating degradation of expressed TEs, PIWI proteins also initiate DNA methylation at loci of transposons (Aravin et al., 2008; Kuramochi‐Miyagawa et al., 2008). While the function of prepachytene piRNAs in fetal germ cells is quite well‐established, the function of pachytene piRNAs remains less explored.

#### 2.1.2 Pachytene piRNAs

In contrast to the fetal/prepachytene piRNAs, the majority of piRNAs found in the adult testis are not antisense to TEs and represent the pachytene piRNAs, which seem to be engaged in the regulation of transcript degradation and translation during the late and postmeiotic stages of spermatogenesis (Dai et al., 2019; Gou, Dai, Yang, et al., 2014; Ozata et al., 2019). In elongating spermatids, transcripts with many pachytene piRNA target sites in the 3′UTR appear to be degraded faster than transcripts without piRNA target sites in the 3′UTR (Gou, Dai, Yang, et al., 2014). In contrast, the translation of other transcripts was reduced when specific piRNAs or their 3′UTR binding sites were mutated, indicating some piRNAs also engage in translational activation of specific transcripts (Dai et al., 2019). Hence, the pachytene piRNAs seem to regulate transcript availability in meiotic/postmeiotic germ cells and consequently to serve a different biological purpose than the fetal/prepachytene piRNAs.

Several studies on pachytene piRNAs and their associated PIWI proteins indicate that the presence of functional piRNAs is essential for spermatogenesis and piRNAs thereby affect male fertility (Beyret & Lin, 2011; Carmell et al., 2007; Kuramochi‐Miyagawa et al., 2004; Ozata et al., 2019). We have recently demonstrated that the presence of mature piRNAs and a functional 3′ exonuclease, PARN‐like ribonuclease domain containing 1 (PNLDC1, also known as “Trimmer”), are essential for spermatogenesis in men (Nagirnaja et al., 2021). In fact, emerging data indicate that most proteins involved in the piRNA biogenesis affect male fertility (Kherraf et al., 2022).

### 2.2 piRNA biogenesis

piRNAs are produced from long piRNA precursor transcripts containing many piRNAs (Figure 1). piRNA precursor transcripts are processed into mature piRNAs in a complex process involving multiple factors such as PIWI and Tudor domain‐containing proteins (Sun et al., 2021). Two principally different pathways of biogenesis have been described (Kim et al., 2009; Ozata et al., 2019). Pachytene piRNAs are mainly produced through the primary biogenesis pathway (Figure 1), but the prepachytene piRNAs also take advantage of the ping‐pong (also referred to as the secondary) biogenesis pathway, which allows great amplification of specific piRNAs to target expressed TEs (Sun et al., 2021). Because the ping‐pong amplification prevents TEs from spreading in the genome, this has also been regarded as part of the adaptive immune response (Haase, 2016).

Interestingly, transcription of pachytene piRNA precursor transcripts seems to be controlled by a single master transcription factor, MYB proto‐oncogene like 1 (MYBL1) and uses RNA polymerase II (RNA pol II) (Bolcun‐Filas et al., 2011; Li et al., 2013). The precursor transcripts are transported to the cytoplasm and accumulate in germ granules, the intermitochondrial cement between the mitochondria, and later during spermatogenesis in the chromatoid body (Gomes Fernandes et al., 2018; Wang et al., 2020). In the chromatoid body, endonucleolytic cleavage of precursor transcripts produces pre‐piRNAs, which undergo further maturation by exonucleolytic trimming by PNLDC1 and 2′‐*O*‐methylation by HEN methyltransferase 1 (HENMT1) (Ding et al., 2017; Gainetdinov et al., 2021; Gou, Dai, & Liu, 2014; Nishimura et al., 2018).

## 3 PIRNAS AND FERTILITY

While the importance of piRNAs for spermatogenesis in mice and *Drosophila* has been known for many years, not much is known about the role of piRNAs in humans. A few studies in humans have been published and, not surprisingly, they reveal that pathogenic mutations in genes of the piRNA biogenesis pathway cause male infertility (Kherraf et al., 2022; Nagirnaja et al., 2021).

### 3.1 Animal studies

Several studies in mice have demonstrated that PIWI proteins and other proteins involved in the piRNA biogenesis pathways are essential for spermatogenesis (Ozata et al., 2019). Studies using mouse models with knockout of genes encoding piRNA biogenesis‐associated proteins nearly all report an arrest of germ cells at various stages of spermatogenesis. Similar phenotypes are observed in the mouse models lacking expression of *Mili*, *Miwi2*, *DExD‐box helicase Mov10‐like‐1* (*Mov10l1*), *Tudor and KH domain‐containing protein* (*Tdrkh*), and *Pnldc1* including upregulation of TEs, impaired spermatogenesis and reduced DNA methylation (Ding et al., 2017; Frost et al., 2010; Kuramochi‐Miyagawa et al., 2004, 2008; Manakov et al., 2015; Nishimura et al., 2018; Saxe et al., 2013; Zhang et al., 2017). More specifically for spermatogenesis, *Miwi* knockout causes spermatogenic arrest in early round spermatids while knockout of *Mili* causes spermatogenic arrest in spermatocytes (Deng & Lin, 2002; Kuramochi‐Miyagawa et al., 2004). Spermatogenic arrest at the meiotic zygotene stage is observed in the *Tdrkh* knockout mouse and meiotic arrest at early prophase I in the Mov10l1 knockout model (Frost et al., 2010; Saxe et al., 2013). Finally, knockout of *Pnldc1* causes both meiotic and postmeiotic arrest in late pachytene spermatocytes, and round and elongated spermatids (Ding et al., 2017; Nishimura et al., 2018; Zhang et al., 2017).

Only a few studies have investigated which piRNAs are essential for spermatogenesis. A recent study deleted several piRNA clusters in mice and found that mice lacking the conserved pachytene piRNA cluster on chromosome 6 (pi6) show defects in sperm capacitation and maturation (Wu et al., 2020). Besides the pi6 cluster, mice lacking the pi18 cluster show defects in the growth of the sperm acrosome (Choi et al., 2021). However, there is still a significant knowledge gap regarding the role of PIWI proteins, piRNA biogenesis‐associated proteins, and specific piRNA clusters in human spermatogenesis.

### 3.2 Studies in humans

Quite surprisingly, only a few of the piRNA genes known to cause male sterility in mice have been linked to spermatogenic impairment in men (Table 2). In fact, no piRNA‐related genes are listed among male infertility genes in two recent comprehensive reviews on the topic (Houston et al., 2021; Oud et al., 2019). Very recently we have, nevertheless, described that piRNAs and proper processing of piRNAs are essential for human spermatogenesis by analyzing testicular piRNAs from men with pathogenic mutations in *PNLDC1* (Nagirnaja et al., 2021). Four men with a defective *PNLDC1* showed testicular down‐regulation of the piRNA proteins PIWIL1, PIWIL4, MYBL1, and TDRKH, and their piRNA profiles indicated fewer and longer piRNAs (Nagirnaja et al., 2021). Hence, functional data support that mature piRNAs are needed for human spermatogenesis. In addition to PNLDC1, a study has identified pathogenic mutations in *TDRD7* in two brothers with nonobstructive azoospermia (NOA) from a consanguineous family (Tan et al., 2019) and mutations in *TDRD9* have been identified as the likely cause of NOA in four men from a large consanguineous Bedouin family (Arafat et al., 2017). However, single‐case and family studies often lack the power to show that this particular variant in fact is causal and studies also often miss out to show the effect of such variants on the testicular piRNA profile. More comprehensive studies were performed on PIWIL1 (Gou et al., 2017) where human data were combined with mouse models. Three Han Chinese patients with NOA showed several heterozygous mutations in the ubiquitination D‐box element of *PIWIL1* (Gou et al., 2017). In mice, these D‐box mutations displayed deficiency in ubiquitination and degradation of PIWIL1 causing spermatogenic failure in late spermatids (Gou et al., 2017). However, studies in larger cohorts have questioned whether mutations in *PIWIL1* are relevant (Oud et al., 2021). In a recent study, whole‐exome sequencing of unrelated men with NOA revealed homozygous loss‐of‐function mutations in *TDRKH*, *TDRD9*, and *HENMT1* in four men (Kherraf et al., 2022). In addition, a missense mutation in *HENMT1* was observed in another man (Kherraf et al., 2022). Mutations in these genes could cause deficient piRNA biogenesis leading to accumulation of immature piRNAs and spermatogenic arrest. Alterations in piRNA expression profiles have also been observed in seminal plasma from infertile men, where men with asthenozoospermia or azoospermia had lower piRNA levels than fertile controls (Hong et al., 2016), and in testicular tissue from men undergoing TESE, where men with unsuccessful TESE had lower levels of piRNAs than men with successful TESE (Cao et al., 2018). Taken together, pachytene piRNAs and their associated proteins seem to be essential for fertility in men, but much more evidence is needed to leverage information on the role of piRNAs in testicular function, male reproductive endocrinology, and spermatogenesis.

**TABLE 2:** Mutations in piRNA biogenesis‐associated protein genes that cause spermatogenic arrest in men

Genes (references)	Functional consequence of genetic variant(s)	Clinical presentation	Testicular histology	ClinGen evidence level^a^
*PNLDC1* (Nagirnaja et al., 2021)	Patient 1: stop‐gain variant Patient 2: missense variant Patient 3: two compound heterozygous mutations Patient 4: canonical splice acceptor site variant	NOA	Spermatogenic arrest	Strong
*TDRD7 (*Tan et al., 2019 *)*	Two brothers (consanguineous family): Loss‐of‐function variants	NOA	Spermatogenic arrest (maturation arrest)	Limited
*TDRD9* (Arafat et al., 2017; Kherraf et al., 2022)	Five men (Bedouin family): a frame‐shift variant in exon 5	NOA	Spermatogenic arrest (maturation arrest)	Limited
	Patient 0080^a^ and 0279: frame‐shift variants hypospermatogenesis^a^	NOA	Spermatogenic arrest (meiosis) *only for Patient 0279*	N/A
*TDRKH (*Kherraf et al., 2022 *)*	Patient 0110: stop‐gain variant	NOA	Spermatogenic arrest (meiosis)	N/A
*HENMT1 (*Kherraf et al., 2022 *)*	Patient 0272: stop‐gain variant	NOA	Spermatogenic arrest (meiosis)	N/A
*PIWIL1 (*Gou et al., 2017, 2021; Oud et al., 2021 *)*	Three Han Chinese patients: missense variants (Gou et al., 2017). Not identified in a larger cohort (Oud et al., 2021)	Idiopathic azoospermia	N/A	N/A

Abbreviations: NOA, Nonobstructive azoospermia; N/A, Not available.

^a^ Harrison et al. (2019) and Richards et al. (2015).

## 4 PERSPECTIVES

piRNAs have been shown to be essential for spermatogenesis in animals and although piRNAs appear to mainly be specific to germ cells, knowledge about their potential role in health and disease is expanding. Studies have shown that piRNAs may play crucial roles both in tumor suppression and tumorigenesis (Liu et al., 2019), and one could imagine that targeting of piRNAs represents a new treatment option as the piRNAs show subgroup specific expression profiles in tumor tissue. Furthermore, the piRNA expression profiles correlate with clinical features related to specific types of tumors (Martinez et al., 2015).

In Table 3, we list studies describing piRNAs in tissues or biofluids not in direct contact with germ cells. Several studies have identified piRNAs in circulation but it is difficult to imagine what biological functions the piRNAs could have in circulation (Foers et al., 2020; Freedman et al., 2016; Kumar et al., 2019) (Table 3). Our group have also described the presence of piRNAs in circulation and found that these were generally present at lower levels among men with impaired testicular function (Kumar et al., 2019), indicating that they could be a proxy for testicular function. The same has been observed in seminal plasma where piRNAs were present at lower levels in men with low semen quality (Hong et al., 2016). piRNAs have also been reported in serum samples from females (Foers et al., 2020), and recent studies have shown that piRNAs are essential for mammalian female fertility (Guan & Wang, 2021). The function of piRNAs outside the germline and the validity of such findings are however questionable. It is difficult to discern whether circulating piRNAs represent leakage from germ cells or if the piRNAs are actively secreted by the germ cells, and whether such piRNAs could mediate biological communication from the testes to other organs. Most studies build on small RNA sequencing, and several pitfalls exist in the experimental procedures as well as the bioinformatics approaches. First, major differences exist between the ability of different library preparation kits to detect piRNAs mainly due to their 2′‐*O*‐methylation, which makes 3′ adapter ligation difficult (Dard‐Dascot et al., 2018). This problem has been addressed by Munafó and Robb, who published a method to overcome the 3′ bias during cDNA synthesis by describing several optimization steps during cDNA synthesis (Munafó & Robb, 2010). Secondly, Tosar et al. described that a subset of piRNAs present in different piRNA databases has 100% sequence identity with other noncoding RNAs and lacked the classical 5′ uridine commonly found in piRNAs. This proposes that some annotated piRNAs are classified as piRNAs, although they are actually fragments of ncRNAs like transfer RNAs (tRNAs), ribosomal RNAs (rRNAs), Y RNAs, small nuclear RNAs (snRNAs), or small nucleolar RNAs (snoRNAs) (Tosar et al., 2018). The misclassified piRNAs in the studied databases (RNAdb 2.0 and piRBase) represented 0.23% and 0.85% of the total number of included sequences in the databases, respectively. Although the percentage of misclassified piRNAs in these databases seem low, there was a clear overlap between the misclassified piRNAs and the highly abundant piRNAs found in plasma and other somatic tissues (Tosar et al., 2018) (Table 3). This makes the misclassification of small RNA fragments as piRNAs a problem, since there is a rather large number of piRNAs reported to be present outside of the gonads that are not really piRNAs. Also, this question both the role and presence of piRNAs outside the gonads. Proteins involved in the piRNA pathway have also been identified outside of the gonads in, for example, cancer cells, but any potential function of the proteins appeared independent from the piRNA pathway, since piRNA expression was absent (Genzor et al., 2019). Hence, small RNA sequencing data, and especially data originating from tissues or biofluids without germ cells, should be interrogated with caution to ensure that the reads are piRNAs and not fragments of other ncRNAs.

**TABLE 3:** Extragonadal tissues/biofluids where PIWI‐interacting RNAs (piRNAs) have been identified

	Sample type	References	Overlap with Tosar et al.^a^
Circulation	Human blood plasma	Males: (Freedman et al., 2016) Females: (Foers et al., 2020)	Freedman: Partially Foers: Partially
Saliva	Sputum sample	Bahn et al. (2015)	Partially
Urine	Urinary sample	Yeri et al. (2017)	Yes
Brain	Prefrontal cortex	Qiu et al. (2017) and Roy et al. (2017)	Qiu: Partially Roy: Yes
Heart	Cardiomyocytes	La Greca et al. (2020)	No
Tumors	Bladder Kidney, liver, pancreas Lung Ovary, endometrium, cervix, prostate, Breast Thyroid Hematological Glioblastoma Colon, gastric Squamous cell carcinoma, fibrosarcoma	Liu et al. (2019) Martinez et al. (2015)	Liu: Partially Martinez: Partially

Abbreviations: No, 0% overlap; Partially, some overlap; Yes, 100%.

^a^ Overlap between piRNAs found in reference articles with the list of fragments of other RNAs the size of piRNAs in Tosar et al.

## 5 CONCLUSION

piRNAs are a special subtype of sncRNAs that appear specific to germ cells. In fetal germ cells, the main function of piRNAs seems to be the regulation of TEs, while in the adult testis, piRNAs regulate the degradation and translation of the transcripts that are crucial for spermatogenesis. Most piRNA components have been shown to be essential for spermatogenesis in animals, but there is still a lack of evidence for their function in humans. Surprisingly, piRNAs have also been identified in human extragonadal tissues and biofluids but their biological and physiological function there remains uncertain. Future research will further elucidate whether extragonadal piRNAs can be used as biomarkers for testicular function and whether such piRNAs carry physiological information.

## AUTHOR CONTRIBUTIONS

**Gülizar Saritas:** Conceptualization (supporting); visualization (lead); writing – original draft (equal); writing – review and editing (equal). **Ailsa Maria Main:** Conceptualization (supporting); data curation (supporting); writing – original draft (equal); writing – review and editing (equal). **Sofia Boeg Winge:** Conceptualization (supporting); writing – review and editing (equal). **Nina Mørup:** Conceptualization (supporting); data curation (equal); investigation (equal); writing – review and editing (equal). **Kristian Almstrup:** Conceptualization (lead); funding acquisition (equal); supervision (equal); writing – original draft (supporting); writing – review and editing (equal).

## FUNDING INFORMATION

The authors are grateful for the financial support given by the Novo Nordisk Foundation (grant numbers 0069969 and 0069913 to Kristian Almstrup), the Capital Region (to Nina Mørup), the Independent Research Fund Denmark (grant number: 1030‐00381B to Kristian Almstrup), and the Svend Andersen Foundation (grant number 84‐A.08 to Kristian Almstrup).

## CONFLICT OF INTEREST

The authors have declared no conflicts of interests for this article.

## RELATED WIREs ARTICLES

Small noncoding RNAs and male infertility

## Data Availability

Data sharing is not applicable to this article as no new data were created or analyzed in this study.
